# Can Activation of Acetylcholinesterase by β-Amyloid Peptide Decrease the Effectiveness of Cholinesterase Inhibitors?

**DOI:** 10.3390/ijms242216395

**Published:** 2023-11-16

**Authors:** Irina V. Zueva, Elmira A. Vasilieva, Gulnara A. Gaynanova, Andrey V. Moiseenko, Anna D. Burtseva, Konstantin M. Boyko, Lucia Ya. Zakharova, Konstantin A. Petrov

**Affiliations:** 1 Arbuzov Institute of Organic and Physical Chemistry, Federal Research Center “Kazan Scientific Center of the Russian Academy of Sciences”, Arbuzov Str., 8, 420088 Kazan, Russia; zueva.irina.vladimirovna@gmail.com (I.V.Z.); lucia@iopc.ru (L.Y.Z.); 2 Faculty of Biology, Lomonosov Moscow State University, Leninskie Gory, 1–12, 119991 Moscow, Russia; 3 Bach Institute of Biochemistry, Research Center of Biotechnology of the Russian Academy of Sciences, Leninsky Prospect, 33/2, 119071 Moscow, Russia; a.burtseva@fbras.ru (A.D.B.); kmb@inbi.ras.ru (K.M.B.); 4 Landau Phystech School of Physics and Research, Moscow Institute of Physics and Technology, Institutsky Lane, 9, Dolgoprudny, 141700 Moscow, Russia; 5 Institute of Fundamental Medicine and Biology, Kazan Federal University, 18 Kremlyovskaya Str., 420008 Kazan, Russia

**Keywords:** β-amyloid peptide, cholinesterase, non-essential enzyme activators

## Abstract

A central event in the pathogenesis of Alzheimer’s disease (AD) is the accumulation of senile plaques composed of aggregated amyloid-β (Aβ) peptides. The main class of drugs currently used for the treatment of AD are the acetylcholinesterase (AChE) and butyrylcholinesterase (BChE) inhibitors. In this study, it has been shown that Aβ augmented AChE activity in vitro, maximum activation of 548 ± 5% was achieved following 48 h of incubation with 10 μM of Aβ_1–40_, leading to a 7.7-fold increase in catalytic efficiency. The observed non-competitive type of AChE activation by Aβ_1–40_ was associated with increased V_max_ and unchanged K_m_. Although BChE activity also increased following incubation with Aβ_1–40_, this was less efficiently achieved as compared with AChE. Ex vivo electrophysiological experiments showed that 10 μM of Aβ_1–40_ significantly decreased the effect of the AChE inhibitor huperzine A on the synaptic potential parameters.

## 1. Introduction

Alzheimer’s disease (AD) is a genetic and sporadic brain disorder that causes the loss of intellectual and social skills [1]. AD is morphologically defined by the presence of β-amyloid-containing plaques and tau-containing neurofibrillary tangles, which correlate with neuronal degeneration and brain atrophy [2]. Unfortunately, the causes of the disease are currently unknown [3]. Therefore, current therapies are largely symptomatic and aimed at delaying the moment of complete disability of patients [4].

Symptomatic AD therapies are generally based on the “cholinergic hypothesis”. Observed characteristics of AD neuropathology include the massive loss of basal forebrain cholinergic neurons, resulting in cholinergic deficits in various brain regions [5,6,7]. These observations led to the introduction of acetylcholinesterase (AChE) and butyrylcholinesterase (BChE) inhibitors as a primary treatment for AD. These enzymes deactivate the neurotransmitter acetylcholine (ACh), partially inhibit cholinesterases (ChE) and can compensate for the cholinergic deficit. In most patients, treatment with ChE inhibitors has a positive effect during the early stages of the disease [8].

The main pathological hallmark of AD is a progressive production of oligomerized amyloid-β (Aβ) peptides, followed by the formation of Aβ plaques. Accumulation of oligomerized Aβ may trigger or contribute to the process of neurodegeneration. Oligomerized Aβ causes a wide range of neurotoxic effects, such as impaired synaptic transmission and plasticity, and mitochondrial dysfunction, etc. [9,10,11,12,13,14,15].

Many studies have attempted to determine the specific cell-surface receptors for Aβ and its oligomers [16]. It is important to note that AChE—the enzyme whose inhibition is used for the treatment of AD—can act as a “pathological chaperone” during Aβ oligomerization. AChE has been shown to promote Aβ oligomerization by forming a complex with growing fibrils [17,18,19,20]. AChE also shifts the balance towards the predominance of Aβ toxic forms and potentiates neurodegenerative changes induced by the Aβ peptide [21,22,23]. Thus, Aβ–AChE interactions can participate in the amplification of AD progression. From this point of view, it is also of interest to consider whether Aβ peptide can affect the catalytic activity of AChE. However, despite the ample evidence of Aβ–AChE interactions, the direct effect of Aβ oligomers on the enzyme activity has never been studied. Understanding the intricate relationship between Aβ peptides and ChE activity can be crucial to developing effective therapeutic strategies such as development of new ChE inhibitors and identification of alternative targets for medical intervention.

Thus, the current study set out to test the hypothesis that the interactions between ChE and Aβ can modulate ChE catalytic activity. It is shown that incubation of Aβ_1–40_ with AChE or BChE increases the catalytic activity of both enzymes. Moreover, the effect of activation of AChE by Aβ is much more pronounced than for BChE. Ex vivo electrophysiological experiments demonstrated that the application of Aβ significantly reduced the effect of the AChE inhibitor huperzine A on the parameters of excitatory synaptic potentials.

## 2. Results

### 2.1. Aβ_1–40_ Increases Catalytic Activity of AChE and BChE In Vitro

**Figure 1:**
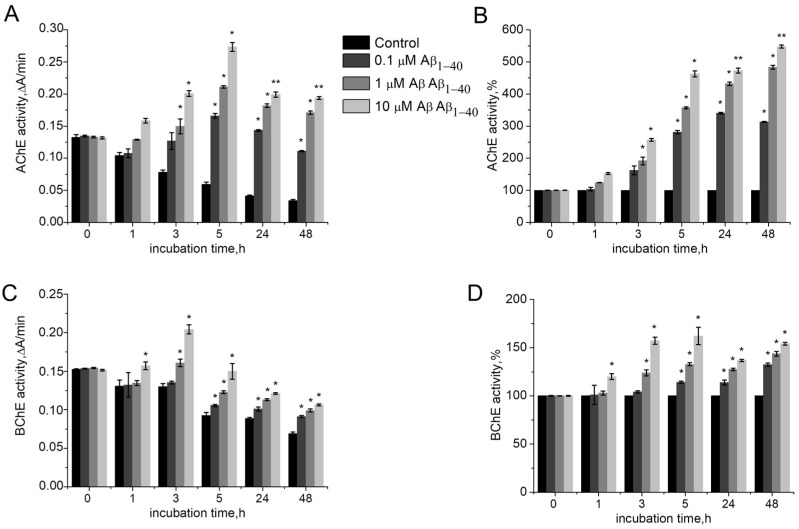
Activity of human AChE (**A**) and human BChE (**C**) in the absence and presence of 0.1–10 μM Aβ_1–40_ at 412 nm in 0.1 M phosphate buffer (pH 8.0 for AChE and pH 7.0 for BChE) at 25 °C. Identical activities of human AChE (**B**) and BChE (**D**), expressed as a percentage of the control level. Data are presented as mean ± SEM. *—difference to control is statistically significant at *p* ≤ 0.05; **—at *p* ≤ 0.01. Statistical analysis was carried out using the Mann–Whitney test (n = 3).

To evaluate the effect of Aβ_1–40_ on ChE activity, AChE or BChE were incubated in the presence of various concentrations of Aβ_1–40_ (1–10 μM) for 1 h, 3 h, 5 h, 24 h, and 48 h. Aβ_1–40_ was assessed to enhance AChE and BChE activity beyond an existing basal level (Figure 1A,C). Thus, Aβ_1–40_ can be considered as a non-essential enzyme activator. The AChE activity shows a trend of increasing over time at each tested concentration of Aβ_1–40_ (Figure 1B). The most significant AChE activation effect was detected for 10 µM Aβ_1–40_ after 48 h of incubation: here, AChE activity increased 5.4 times (*p* = 0.007) as compared to AChE activity without Aβ peptide (Figure 1B). The AChE activation plateau was reached 24 h after incubation with Aβ_1–40_: no significant increase in activity after 48 h of incubation as compared with 24 h incubation was observed (Figure 1B).

Aβ_1–40_ was shown to activate BChE less effectively than AChE. A significant increase in BChE activity by 0.6 times (*p* = 0.041) was observed after 3 h of incubation with Aβ_1–40_ (Figure 1D). During the next few hours of incubation, the activity did not change significantly.

It should be noted that despite AChE and BChE activity being decreased over the incubation time (Figure 1A,C), in general the level of enzyme activity during the experiment was higher than at the beginning of the incubation period, which indicates an activating, rather than a stabilizing property of Aβ_1–40_.

It has been reported that peptides self-assembled into amyloids (other than Aβ) itself can catalyze hydrolysis of esters [24]. The increase in esterase activity can be assumed to occur due to hydrolysis of the substrate by aggregated Aβ. To assess whether Aβ_1–40_ itself can catalyze hydrolysis of substrates for AChE and BChE, the rate of acetylthiocholine (ATC) and (butyrylthiocholine) (BTC) hydrolysis was assessed without enzymes. However, no direct effect of Aβ_1–40_ on the ATC and BTC hydrolysis was observed. In the presence of Aβ_1–40_ aggregated for 24 h, the rate of ATC and BTC spontaneous hydrolysis was not observed to change. Thus, increased esterase activity occurs precisely as a result of the effect of Aβ on the catalytic properties of ChE.

**Figure 2:**
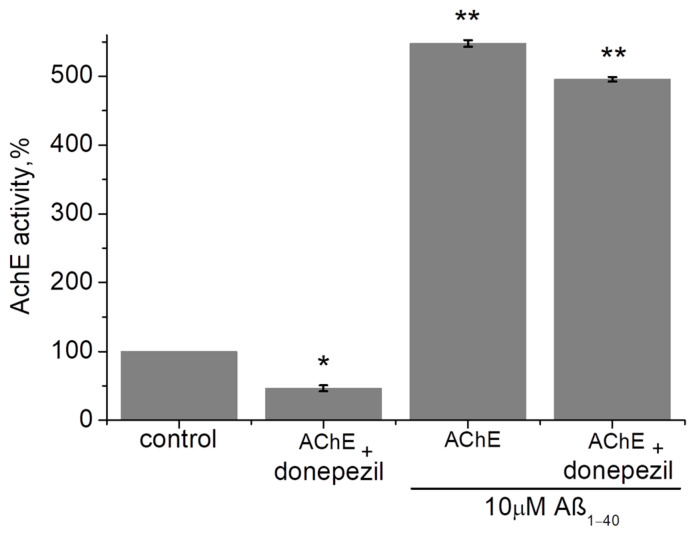
The effect of Aβ_1–40_ (10 µM) on the AChE inhibited by donepezil was measured after 24 h of incubation at 412 nm in 0.1 M phosphate buffer (pH 8.0) at 25 °C. Data are presented as means ± SEM. *—the difference with the control is statistically significant at *p* ≤ 0.05; **—at *p* ≤ 0.01. Statistical analysis was carried out using the Mann–Whitney test (n = 3).

The inhibitors of ChE are widely used during symptomatic AD treatment. However, it can be assumed that the effect of ChE activation can interfere with the effect of ChE inhibition. The effect of Aβ_1–40_ on the inhibited AChE was studied (Figure 2). Initial enzyme activity was taken as 100%. AChE was inhibited by 10 nM of donepezil to 46.5 ± 4.5% (*p* = 0.045). It was shown that incubation of the inhibited AChE with Aβ_1–40_ (10 µM) within 24 h significantly increased the AChE activity up to 495.6 ± 3.5% (*p* = 0.009). It is important to note that no significant inhibitory effect of donepezil was observed compared to AChE incubated with Aβ_1–40_ alone (without donepezil). Thus, the effect of AChE activation by Aβ_1–40_ may counteract the effect of AChE inhibition.

### 2.2. Determination of ChE Kinetic Parameters after Incubation with Aβ_1–40_

**Scheme 1:**
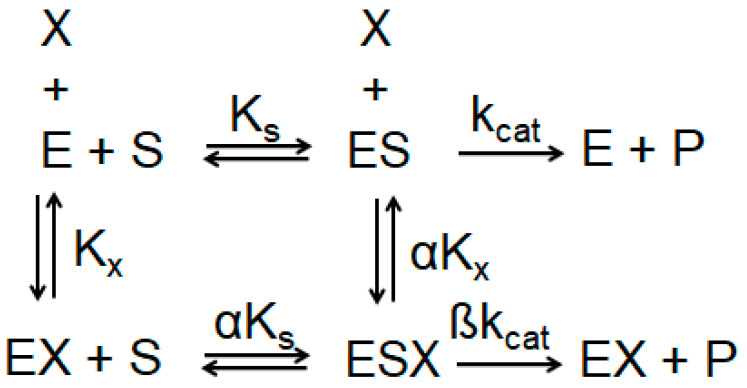
General scheme of ChE modifier. X—enzyme modifier; E—ChE; ES—complex of ChE with substrate; K_x_—equilibrium dissociation constant for activator binding to free ChE; K_s_—equilibrium dissociation constant for substrate binding to free ChE; k_cat_—rate for the product-generating chemical step; α—value determining the relative affinity for X binding to free ChE; β—factor determining the overall rate enhancement induced by the modifier.

The mechanism of action of Aβ_1–40_ on AChE and BChE was studied. The activity of AChE and BChE with 1–10 μM Aβ_1–40_ was assessed after 24 h of incubation with the peptide, at which point ChE activation reaches a plateau. The kinetic parameters of the reaction of hydrolysis of the ATC and BTC (V_max_, K_m_) without Aβ_1–40_, as well as after incubation with 1–10 μM Aβ_1–40_, was measured. According to the general scheme of an enzyme modifier (Scheme 1) [25], the type of activator and mechanism of enzyme activation can be determined by the ratio of the α and β values.

**Figure 3:**
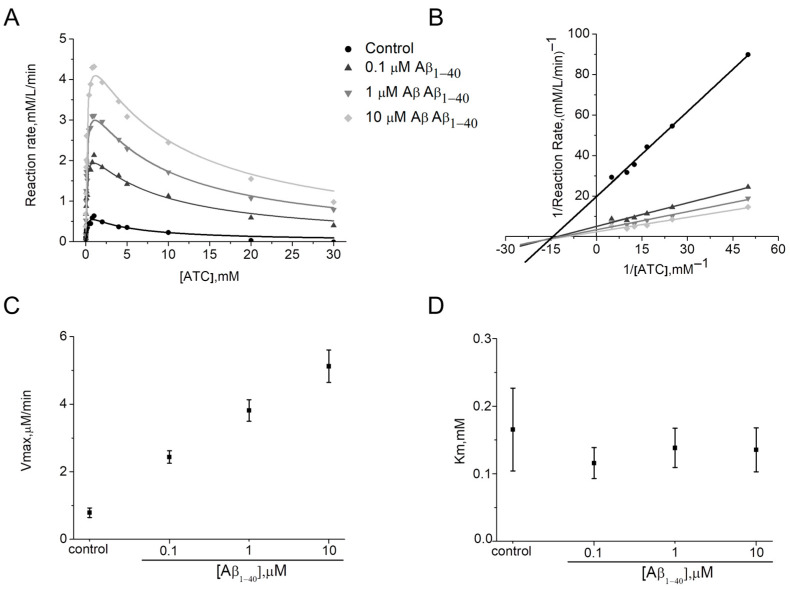
Demonstration of the type of activation of human AChE by Aβ_1–40_ at 412 nm in phosphate buffer 0.1 M, pH 8.0 at 25 °C. Data are expressed in the form of representative kinetic curves of the reaction rate of human AChE with ATC (**A**) and in the form of a representative Lineweaver–Burk plot for ATC concentrations below 1 mM (**B**). Changes of kinetic constants V_max_ (**C**) and K_m_ (**D**) were determined by non-linear fitting of reaction rate curves to Equation (2). Data are presented as means ± SEM (n = 3).

For AChE, K_m_ in the presence of Aβ_1–40_ was unchanged (α = 1) (Figure 3A,D, Table 1). At the same time, increases of V_max_ (β > 1) (Figure 3A,C; Table 1) indicating enhanced substrate affinity to the AChE active site was observed. Thus, for 10 μM Aβ_1–40_ a 6.3-fold increase in V_max_ compared to the control was shown. Therefore, for human AChE the activation type can be considered as non–competitive V–type. Activators of this type potentially enhance the kinetic rate by realigning the kinetic machinery; here, the rate of the chemical step is increased without affecting the substrate binding [26]. It can be stated that Aβ_1–40_ may bind before and after the ATC binding with equal affinity to the free AChE and complex of AChE with ATC. The intersection of the Lineweaver–Burk plots (Figure 3B) in the absence and presence of 0.1–10 μM Aβ_1–40_ confirms that K_m_ does not change. In addition, gradual increases up to 2.4 times in K_ss_ values with changing Aβ_1–40_ concentrations were shown (Table 1).

For simplicity, the overall rate can be described by a single first-order rate constant k_cat_ proportional to the V_max_ values (i.e., k_cat_ = V_max_/[ChE]) [27] and the kinetic efficiency of ChE in the presence of Aβ is best defined by the second-order hydrolysis rate constant k_cat_/K_m_ [28]. Table 1 shows the estimated k_cat_ and k_cat_/K_m_ ratio for AChE.

Hydrolysis of ATC by human AChE is rapid; here, the second-order hydrolysis rate constant is equal to 3.1 ± 1.1 × 10^9^ M^−1^ min^−1^. The increase in this ratio with the presence of 0.1–10 μM Aβ_1–40_ in the medium indicates an increase in the kinetic efficiency of the AChE (Table 1). Thus, it is shown that incubation of the AChE with 10 μM Aβ_1–40_ leads to a catalytic efficiency 7.7-fold greater than that of AChE in the control.

**Figure 4:**
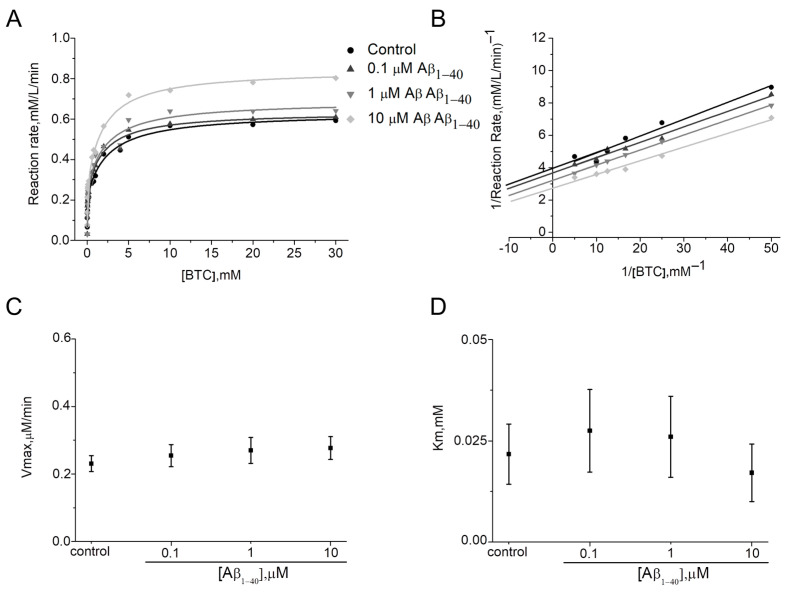
Demonstration of the type of activation of human BChE by Aβ_1–40_ at 412 nm in phosphate buffer 0.1 M, pH 7.0 at 25 °C. Data are expressed in the form of representative kinetic curves of reaction rate of human BChE with BTC (**A**) and in the form of representative Lineweaver–Burk plots for BTC concentrations below 1 mM (**B**). Change in kinetic constants V_max_ (**C**) and K_m_ (**D**) were determined by non-linear fitting of reaction rate curves to Equation (2). Data are presented as means ± SEM (n = 3).

Determining the type of activation for BChE by V_max_ and K_m_ was complicated due to the small percentage of activation (maximum 154 ± 1.5%). A minor decrease in K_m_ and an increase in V_max_ were observed (Figure 4A,C,D; Table 2). However, K_m_ values have no defined trend, and according to the Lineweaver–Burk plot for BTC concentrations below 1 mM, a mixed type of activation can be assumed (Figure 4B). Thus, Aβ_1–40_ is able to bind to BChE before, or after BTC with varying affinity. In addition, there were no changes in K_ss_ values with increasing Aβ_1–40_ concentrations (Table 2).

Human BChE demonstrated catalysis of BTC hydrolysis with k_cat_/K_m_ about 1.0 ± 0.3 × 10^9^ M^−1^·min^−1^ (Table 2). However, the catalytic efficiency increased only 1.5 times in the presence of 10 μM Aβ_1–40_ (Table 2).

It is well known that both BChE and AChE display a non-Michaelian behavior at high concentrations (above 1 mM) of substrates with thiocholine esters; however, AChE is inhibited by an excess of ATC [29], while BChE is activated by an excess of BTC [30]. The activator or inhibitory effect resulted from the binding of excess substrate molecules on the enzyme peripheral site (Scheme S1) [31]. It can be noted that while in the presence of Aβ_1–40_, changes in K_ss_ value were shown for AChE (Table 1, Figure 3A), for BChE, no significant change in the K_ss_ with an excess of substrate was observed (Table 2, Figure 4A). Thus, the ability of Aβ_1–40_ to compete with the substrate for binding to the peripheral site of AChE or BChE requires detailed investigation.

### 2.3. Aβ Oligomerization in the Presence of ChE Is Necessary for Enzyme Activation

Aβ is characterized by a high propensity for aggregation [32,33]. There is also evidence that AChE can increase the rate of formation of Aβ fibrils by forming stable Aβ–AChE complexes [17,22,34]. Thus, there is a high probability of AChE activation in the presence of oligomerized Aβ.

Aβ peptide oligomerization can be studied using dynamic light scattering (DLS) and transmission electron microscopy (TEM). Since the fibrillar structures formed by Aβ are not strictly spherical in shape, the DLS data, which are based on a spherical approximation, can be used for qualitatively assessing the structural rearrangements occurring in the solution.

**Figure 5:**
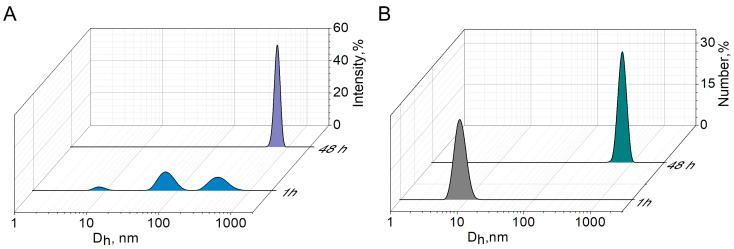
Intensity-averaged size distribution of the Aβ_1–40_ aggregates after 1 and 48 h of incubation in 0.1 M phosphate buffer, pH 8.0, 25 °C (**A**); Number-averaged size distribution of the Aβ_1–40_ aggregates after 1 and 48 h of incubation in 0.1 M phosphate buffer, pH 8.0, 25 °C (**B**).

The number-size distribution of aggregates obtained from the analysis of Aβ_1–40_ solution in a phosphate buffer showed a monomodal distribution with average diameter (D_h_) 8 ± 1 nm after 1 h of incubation (Figure 5B). Aggregates of this size are characterized by the absence of β-structural organization of the protein. The distribution of hydrodynamic diameter by intensity of scattered signal (Figure 5A) is polymodal with a high polydispersity index (PdI 0.7), as also described in [35]. However, from the number-size distribution of aggregates, it can be concluded that there is a predominance of particles with a diameter of less than 10 nm in the solution (Figure 5B). The high polydispersity suggests that Aβ exists as a conglomerate of different conformations [35]. Such a polymodal distribution for Aβ in a buffer solution has previously been demonstrated by other authors [35]; moreover, the rate of Aβ aggregation depends on the temperature at which the peptide was incubated, the highest value being reached at 37 °C [36]. Complete aggregation of Aβ_1–40_ is observed after 48 h at 25 °C as confirmed by the agreement between the values of the D_h_ averaged by the intensity of the scattered signal (Figure 5A), the number of particles (Figure 5B), and the Z-average value (763 nm). The significant reduction in the PDI from 0.7 to 0.3 probably indicates the end of Aβ oligomerization under these experimental conditions.

**Figure 6:**
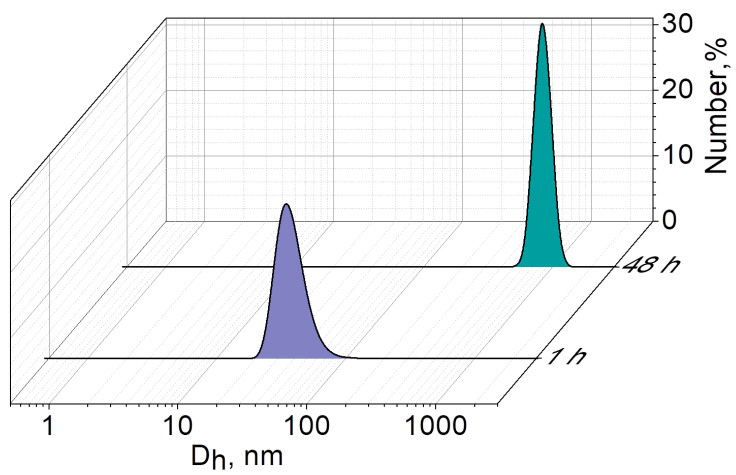
Number-averaged size distribution of aggregates in the mixed Aβ_1–40_/AChE system after 1 and 48 h of incubation in 0.1 M phosphate buffer, pH 8.0, 25 °C.

In the case of the binary Aβ_1–40_/AChE system, aggregates with a D_h_ averaged over the number of particles equal to 39 ± 4 nm are already observed 1 h after mixing the Aβ and AChE (Figure 6). This indicates that AChE promotes the aggregation process of Aβ_1–40_: aggregates larger than 10 nm are not fixed after 1 h in an Aβ_1–40_ solution incubated without AChE. After 48 h of incubation of the Aβ_1–40_/AChE binary system, aggregates with a D_h_ of 845 ± 5 nm are observed. There is a statistically significant (*p* < 0.05) increase in the size of the mixed Aβ_1–40_/AChE composition by ~90 nm compared to pure Aβ_1–40_ aggregates (764 nm). Here, it should be noted that the PdI of Aβ_1–40_ in the presence of AChE is quite low (0.25), which indicates the homogeneity of the system; however, it increases slightly 48 h after mixing the components (from 0.25 to 0.4).

**Figure 7:**
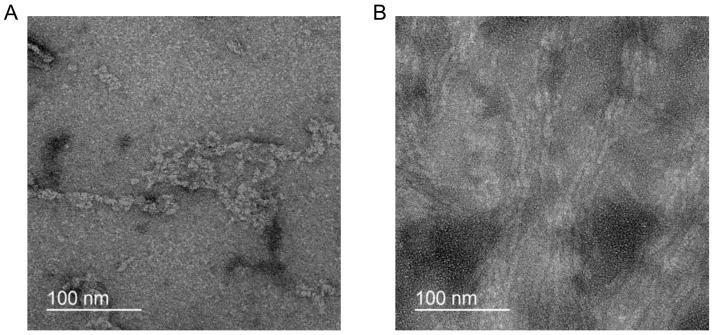
Negative stain TEM images of the Aβ_1–40_ aggregates incubated for 24 h: 10 μM Aβ_1–40_ incubated without (**A**) and with AChE (**B**).

To shed light on the morphology of Aβ_1–40_ aggregates, negative staining TEM for fibrils both with and without AChE was utilized. TEM images of the 10 μM Aβ_1–40_ incubated for 24 h without AChE demonstrated mostly aggregated fuzzy filamentous particles ~7 nm in diameter (Figure 7A). A different situation was found in the binary system, where 10 μM Aβ_1–40_ was incubated for 24 h with AChE. In this case, there were a lot of long intertwined fibrillar particles of about 4 nm diameter (Figure 7B). Noteworthy, negatively stained samples of 10 μM Aβ_1–40_ that were incubated for 3 h with and without AChE did not contain fibrils, possibly due to short incubation times.

**Figure 8:**
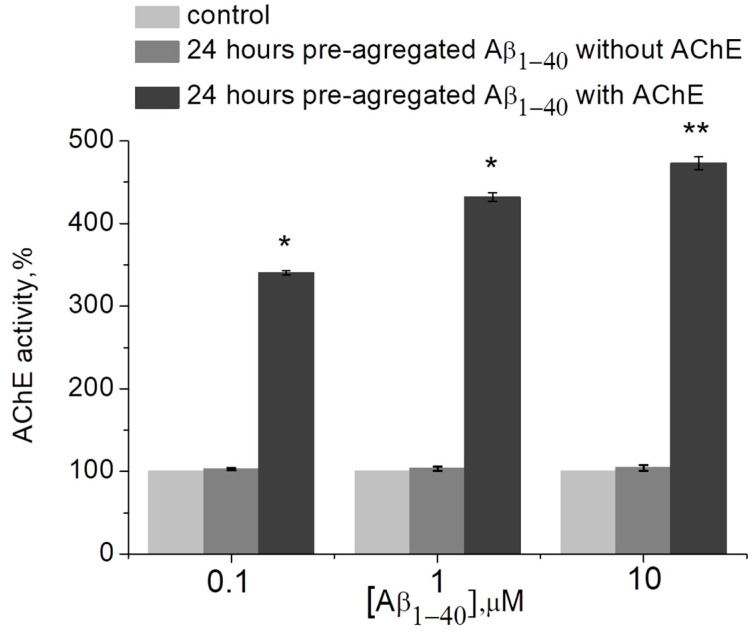
The effect of preliminarily aggregated (within 24 h) Aβ_1–40_ (10 µM) with/or without AChE on the enzyme activity as measured at 412 nm in 0.1 M phosphate buffer (pH 8.0) at 25 °C. Data are presented as means ± SEM. *—the difference with the control is statistically significant at *p* ≤ 0.05; **—at *p* ≤ 0.01. Statistical analysis was carried out using the Mann–Whitney test (n = 3).

The question arises as to whether Aβ aggregation in the presence of AChE is necessary for AСhE activation, or whether enzyme activity can also be increased by Aβ aggregated without AChE. For this reason, the effect of preliminary aggregated Aβ_1–40_ on the activity of AChE was also studied. If Aβ_1–40_ was pre-aggregated in phosphate buffer and AChE was added to the Aβ after 24 h, no increase in AChE activity was observed (Figure 8). Thus, it is probably only by assembling Aβ oligomers on the surface of ChE that the conformational changes in the protein, leading to improved catalytic activity, can be triggered and stabilized.

### 2.4. Activation of Enzyme by Aβ_1–40_ Decreases Efficacy of the Treatment with AChE Inhibitor Ex Vivo

**Figure 9:**
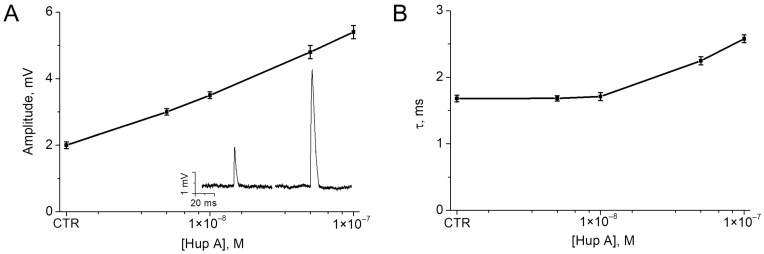
Concentration-dependent effect of huperzine A (Hup A) on amplitude (**A**) and decay time constant (**B**) of miniature end-plate potentials (MEPP) recorded in mouse diaphragm muscle. CTR—control (intact AChE) values. The inset shows representative MEPPs recorded in the control (left MEPP) and after application of 100 nM huperzine A (right MEPP). Mean ± SEM obtained from 20 NMJs, 100 MEPPs in each NMJ were analyzed.

In the final series of experiments, we studied the possibility of activation of AChE, which hydrolyzes endogenous ACh released during synaptic transmission. The neuromuscular junction (NMJ) was selected as a well-studied cholinergic synapse. Inhibition of AChE at the NMJ is known to lead to an increase in the amplitude and duration of end-plate potentials [37]. We used analysis of changes in these parameters to study the effect of Aβ on the activity of synaptic AChE. With partial inhibition of AChE at the NMJ, it can be assumed that activation of residual AChE by Aβ will reduce the effect of the AChE inhibitor on the amplitude and duration of end-plate potentials. Experiments were performed ex vivo on mouse diaphragm muscles. Miniature end-plate potentials (MEPPs) were recorded in the control and following application of the selective AChE inhibitor huperzine A [38]. Huperzine A was shown to increase the amplitude and duration (decay time constant, τ) of MEPP in a concentration-dependent manner (Figure 9).

**Figure 10:**
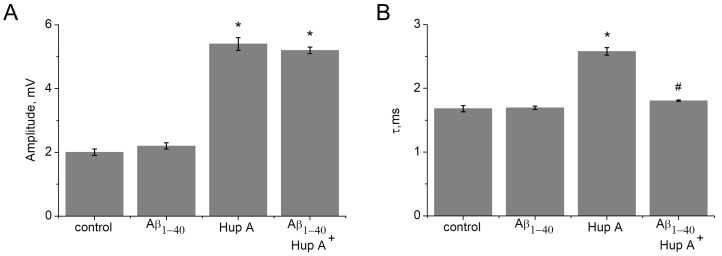
Effect of pre-incubation with Aβ_1–40_ (10 µM) and following AChE inhibition with 100 nM huperzine A (Hup A) on amplitude (**A**) and the decay time constant (**B**) of miniature end-plate potentials (MEPP). *—the difference with the control is statistically significant at *p* ≤ 0.05; #—the difference with the Hup A 100 nM is statistically significant at *p* ≤ 0.05. Statistical analysis was carried out using the Student’s *t*-test. Mean ± SEM obtained from 20 NMJs, 100 MEPPs in each NMJ were analyzed.

A huperzine A concentration of 100 nM, which significantly increases both amplitude and duration of MEPPs, was selected to study the effect of Aβ. After recording MEPPs in the control and after 1 h of incubation of the muscle with Aβ_1–40_ (10 μM), huperzine A (100 nM) was applied. With fully active AChE, incubation with Aβ_1–40_ did not significantly change the amplitude and duration of MEPP (Figure 10). However, the effect of huperzine A following incubation with amyloid on the decay constant was significantly lower. After 1 h of pre-incubation with Aβ_1–40_ (10 µM), the application of huperzine A (100 nM) did not lead to a significant change in the MEPP decay time (Figure 10). Thus, even a fairly short incubation time with Aβ (1 h) is sufficient to detect a statistically significant reduction in the effect of the AChE inhibitor on synaptic transmission.

## 3. Discussion

The present study set out to test the hypothesis that Aβ may influence the catalytic activity of ChE. Our in vitro experiments showed that incubation of AChE with Aβ_1–40_ increases the catalytic efficiency of the enzyme by more than seven times. However, the catalytic efficiency of BChE increases to a lesser extent, by only 1.5 times. The activation of AChE evident after the preceding lag phase correlated with the time of Aβ oligomer formation. According to the kinetics study, Aβ can be considered as a non-essential activator (also termed a positive allosteric modulator) of ChE. This type of activator increases enzyme activity beyond an existing basal level, indicating that the reaction can occur in the absence of the activator, as well as in its presence [25].

The positive modulation of ChE activity has been previously described. It has been shown that the presence of cations (Ca^2+^, Mg^2+^, Mn^2+^) in phosphate buffer increases the activity of ChE in vitro [39]. Allosteric small-molecule AChE activators have also been reported [40]. However, to the best of our knowledge, the phenomenon of ChE activation in the presence of Aβ peptide has not previously been described.

The cholinergic deficit is one of the main hallmarks of AD. Since the discovery of the cholinergic deficit, ChE has been widely investigated in brain tissues obtained post mortem from AD patients [41]. Most of the cortical AChE activity present in post mortem AD brain tissue was found to be co-localized with Aβ deposits [42,43]. This includes both amyloid diffuse deposits and mature senile plaques [44,45]. The diffuse deposition of Aβ together with AChE is particularly interesting since it represents an early step in the development of senile plaques [46]. BChE has also been detected in Aβ plaques, suggesting the possible involvement of the protein in the pathogenesis of AD [47,48,49,50]. Thus, AChE and BChE have been shown to form a complex with senile plaque components in vivo. If activation of AChE by Aβ occurs in vivo, it is possible that this may additionally exacerbate the cholinergic deficit.

The inhibitors of ChE are widely used during symptomatic AD treatment to compensate the cholinergic deficit. Here, it is interesting to note that at least ten-fold higher concentrations of the AChE inhibitor BW284C51 are necessary for complete inhibition of AChE within plaques as compared to the normal enzyme [43]. Small-molecule AChE activators have also been studied as a novel approach in the treatment of organophosphate ChE inhibitor poisoning. The IC_50_ value of paraoxon for AChE has been shown to increase from 20.4 nM to 42.1 nM in the presence of an AChE activator [40]. Thus, the effect of ChE activation can significantly decrease the efficacy of the ChE inhibitor. Our experiments in vitro showed that incubation of AChE with Aβ_1–40_ can decrease the inhibitory effect of donepezil. Our ex vivo experiments showed that Aβ_1–40_ can decrease the effect of the AChE inhibitor huperzine A on decay time of MEPPs at the mouse NMJ. The analysis of MEPP parameters was chosen due to the processes accompanying AChE inhibition at the level of spontaneous ACh release being very well studied. Electrophysiological recordings show that prolonged lifetime of acetylcholine in the synaptic cleft due to AChE inhibition results in an increase in amplitude and duration of MEPPs. It is important to note that the effects of inhibition of synaptic AChE on MEPP parameters depend on the level of inhibition [37,51]. The amplitude increases linearly, while the decay time constant increases exponentially. Until the level of inhibition of AChE activity reaches 90%, only the MEPP amplitude increases. However, when the remaining 10% of AChE activity is inhibited, an exponential increase in the MEPP decay time is observed along with a further slight increase in amplitude (for an example see Figure 9). Thus, in our experiments, a significant decrease in only the decay time constant of MEPP after 1 h of incubation with Aβ_1–40_ following AChE inhibition indicates the achievement of a small (less than 10%) level of synaptic AChE activation. Because of the linear relationship between the level of AChE inhibition and the increase in MEPP amplitude, the effect of such a small augmentation of AChE activity on MEPP amplitude cannot be reliably assessed. This is consistent with the results of experiments on AChE activation in vitro, indicating the need for longer (several days) incubation with Aβ_1–40_ to achieve the highest level of activation.

In conclusion, studies of enzyme modulators, including ChE, remain an important topic of scientific investigation. Based on previous studies of Aβ–ChE interactions and our findings here, we propose a novel mechanism for the modulation of ChE activity involving the Aβ peptide. Further study of the processes associated with the activation of AChE and BChE by Aβ peptides can lead to a better understanding of AD pathogenesis and help to increase efficacy of therapeutic treatments using ChE inhibitors.

## 4. Materials and Methods

### 4.1. Animals

All experiments involving animals were performed in accordance with the guidelines set forth by the European Union Council Directive 2010/63/EU, and conducted in accordance with ARRIVE guidelines, as well as the protocol of experiments approved by the Animal Care and Use Committee of FRC Kazan Scientific Center of RAS (protocol No. 2 from 9 June 2022). Six-week-old CD-1 mice weighing 25–30 g were purchased from the Laboratory Animal Breeding Facility (Branch of Shemyakin-Ovchinnikov Institute of Bioorganic Chemistry, Puschino, Moscow Region, Russia) and allowed to acclimatize to their environment in a vivarium for at least 1 week before experiments. Animals were kept in sawdust-lined plastic cages in a well-ventilated room at 20–22 °C, a 12-h light/dark cycle at 60−70% relative humidity and given ad libitum access to food and water.

### 4.2. Enzymes and Chemicals

Human recombinant Aβ_1–40_ (ultra-pure, HFIP), acetylthiocholine, butyrylthiocholine, 5,5′–dithiobis–(2–nitrobenzoic) acid (DTNB), huperzine A, donepezil, recombinant human acetylcholinesterase and butyrylcholinesterase obtained from human plasma were purchased from Sigma–Aldrich (Sigma–Aldrich Corp., St. Louis, MO, USA). AChE specific activity was 60 U/mL, while BChE specific activity was 450 U/mL based on the Ellman assay with ATC, or BTC as substrate. Dimethyl sulfoxide (DMSO) was purchased from PanReac AppliChem GmbH (Darmstadt, Germany). All other chemicals were of biochemical grade.

### 4.3. Dynamic Light Scattering

Dynamic light scattering experiments were conducted using the Malvern ZetaSizer Nano instrument (Malvern Instruments Ltd., Worcestershire, UK). These experiments utilized a He−Ne laser as the irradiation source, with a wavelength of 633 nm and a power of 4 mW. The analysis of the scattered light involved phase and frequency analysis, which was performed using the dispersion technology software version 5.10. The scattering angle used in all measurements was 173°. Particle size was determined using the Stokes–Einstein Equation (1) for spherical particles.

(1) $$D=\frac{kT}{6\pi \eta R}$$

where D—diffusion coefficient; k—Boltzmann’s constant; T—absolute temperature; η—solvent viscosity; R—hydrodynamic radius. The polydispersity index (PdI) was calculated using cumulative second-order correlation function analysis.

### 4.4. Negative Staining TEM

Copper grids supported by a thin layer carbon film (Lacey C only 300 mesh, Ted Pella, Redding, CA, USA) were glow-discharged for 30 s at 15 mA with a PELCO easyGlow system (Ted Pella, USA). Samples of 3 μL were applied to grids and stained with 1% uranyl acetate in ethanol using a standard protocol. The negatively stained grids were examined using a JEM-2100 (JEOL, Tokyo, Japan) equipped with a Direct Electron DE-20 (Gatan, Pleasanton, CA, USA) camera. The microscope operated at 200 kV with a nominal magnification of 62,000×.

### 4.5. In Vitro Cholinesterases Activity Assay

#### 4.5.1. Determination of ChE Activity In Vitro

All measurements of ChE enzyme activity were performed on a Shimadzu UV–1800 (Shimadzu Co., Tokyo, Japan) spectrophotometer using standard quartz cuvettes of 1 cm-path length in a total volume of 2 mL using Ellman’s assay [52] at 412 nm at 25 °C in 0.1 M phosphate buffer at the optimum pH of AChE (pH 8.0) and BChE (pH 7.0).

Human recombinant Aβ_1–40_ was lyophilized from hexafluoro–2–propanol (HFIP) solution. A 2 mM stock solution of Aβ_1–40_ was prepared in DMSO and stored at −20 °C. Recombinant human AChE or BChE from human plasma were mixed with a solution of Aβ_1–40_ in phosphate buffer. The final Aβ_1–40_ concentration was 0.1, 1, or 10 μM. The final DMSO concentration was 0.5%. After 1–48 h of incubation at 25.0 °C, the enzymatic activity of 100 μL of sample was measured using 0.1 mM DTNB, 1 mM BTC, or 1 mM ATC as a substrate. The absorbance was measured over 2 min. Initial velocities of the reaction were measured in the presence and absence of Aβ_1–40_. An enzyme mixture with 0.5% DMSO was used as a control. The inhibitory effect against ChE of 0.5% DMSO was considered weak [53].

To evaluate the effect of Aβ_1–40_ on the inhibited AChE, enzyme was incubated with 10 nM donepezil to achieve residual enzyme activity ≈ 50%. Then, 10 μM of Aβ_1–40_ was added to the mixture and incubated within 24 h. The enzymatic activity was assessed as described above.

To study the effect of pre-aggregated Aβ on the AChE activity, the activity of the enzyme incubated 24 h with 10 µM Aβ_1–40_ was compared with the activity of the enzyme added to within 24 h, self-aggregated 10 µM Aβ_1–40_. The enzymatic activity was assessed as described above. To assess the effect of aggregated Aβ_1–40_ on the substrate’s hydrolysis, the rate of spontaneous hydrolysis without enzymes was estimated.

#### 4.5.2. Kinetic Parameters Evaluation

0.1–10 μM Aβ_1–40_ was mixed with recombinant human AChE, or BChE from human plasma in 0.1 M phosphate buffer. An enzyme mixture with DMSO was used as a control. Kinetic analysis of ATC and BTC hydrolysis in the absence and presence of Aβ_1–40_ was carried out after 48 h of incubation. The reaction mixture contained 100 μL of sample, 20 μL of 0.01 M DTNB, and 1860 μL of 0.1 M phosphate buffer. The enzymatic reaction was started by adding ATC or BTC. Final substrate concentrations ranged between 0.01 and 30 mM. The active site concentration was 1.5 × 10^−12^ M for AChE and 1.1 × 10^−11^ M for BChE.

The rate of enzymatic reaction was plotted against the substrate concentration. Kinetic parameters K_m_ and V_max_ were determined by non-linear fitting of Equation (2) (Scheme S1) [29].

(2) $$v=\frac{V_{max}}{1+K_{m}/[S]}(\frac{1+b[S]/K_{ss}}{1+[S]/K_{ss}})$$

where K_m_—Michaelis constant, V_max_—enzyme maximum velocity, K_ss_—dissociation constant, b—factor determining the alteration in the catalytic constant k_cat_.

k_cat_ values were determined using the Equation (3).

(3) $$V_{max}=k_{cat}[E]$$

where [E]—ChE concentration.

The type of ChE activation was determined by changing the K_m_ and V_max_ parameters and according to the Lineweaver–Burk plot for substrate concentrations below 1 mM: with an excess of substrates for ChEs, Lineaweaver–Burk plots are non-linear [54].

### 4.6. Ex Vivo Electrophysiology

Mouse hemidiaphragm muscles were isolated from animals killed by cervical dislocation followed by immediate exsanguination. Diaphragm muscles were perfused with oxygenated (95% O_2_, 5%CO_2_) Ringer’s–Krebs’ solution: 154 mM NaCl, 5 mM KCl, 2 mM CaCl_2_, 1 mM MgCl_2_, 5.0 mM HEPES buffer, 11 mM glucose (pH 7.4). Spontaneous miniature end-plate potentials (MEPPs) were recorded at 20–22 °C via standard glass microelectrodes filled with 3 M KCl; the resistance of the electrode was 10–15 MΩ. MEPPs were recorded using an Axoclamp 900A amplifier and digitized using a Digidata 1440A (Molecular Devices, San Jose, CA, USA) with WinWCP V5.5.2 software (John Dempster, University of Strathclyde, Glasgow, UK). Huperzine A and Aβ_1–40_ were dissolved in a Krebs–Ringer solution and applied into the experimental chamber with the muscle.

### 4.7. Statistical Analysis

Data analysis was performed using OriginPro 8.5 software (OriginLab Corporation, Northampton, MA, USA). Each biochemical assay was conducted in triplicate; each experiment was repeated at least three times independently. Data were expressed as mean ± SEM. Statistical analysis was carried out using the Mann–Whitney test. Differences were considered statistically significant at *p* < 0.05. The results of ex vivo electrophysiology experiments are presented as the mean ± SEM of 20 separate end-plates from 4 muscles, obtained from different animals. Data were analyzed using the Student’s *t*-test; differences between the control and experimental values were considered statistically significant at *p* < 0.05.

## Figures and Tables

**Table 1:** Kinetic constants for human AChE, determined in 0.1 M phosphate buffer at pH 8.0 in the presence of Aβ_1–40_ at concentrations within the 0.1–10 μM range for ATC as substrate at 25 °C.

[Aβ_1–40_], μM	K_m_ mM	K_ss_ mM	V_max_ μM/min	k_cat_ min^−1^	k_cat_/K_m_ M^−1^ min^−1^
Control	0.2 ± 0.06	4.1 ± 2.6	0.8 ± 0.1	533,333 ± 66,667	3.1 ± 1.1 × 10^9^
0.1	0.1 ± 0.02	8.1 ± 3.6	2.4 ± 0.2	1,600,000 ± 133,333	1.3 ± 0.7 × 10^10^
1	0.1 ± 0.03	8.5 ± 4.1	3.8 ± 0.3	2,533,333 ± 200,000	1.8 ± 0.7 × 10^10^
10	0.1 ± 0.03	9.7 ± 5.8	5.1 ± 0.5	3,400,000 ± 333,333	2.4 ± 1.1 × 10^10^

**Table 2:** Kinetic constants for human BChE, determined in 0.1 M phosphate buffer at pH 7.0 in the presence of Aβ_1–40_ at concentrations within the 0.1–10 μM range for BTC as substrate at 25 °C.

[Aβ_1–40_], μM	K_m_ mM	K_ss_ mM	V_max_ μM/min	k_cat_ min^−1^	k_cat_/K_m_ M^−1^ min^−1^
Control	0.02 ± 0.01	2.6 ± 0.7	0.2 ± 0.02	20,909 ± 2182	1.0 ± 0.3 × 10^9^
0.1	0.03 ± 0.01	2.0 ± 0.6	0.3 ± 0.03	22,727 ± 3000	0.8 ± 0.3 × 10^9^
1	0.03 ± 0.01	2.3 ± 0.9	0.3 ± 0.04	24,545 ± 3636	0.9 ± 0.4 × 10^9^
10	0.02 ± 0.01	2.0 ± 0.5	0.3 ± 0.03	25,454 ± 2727	1.5 ± 0.4 × 10^9^

## Data Availability

Data available upon request.

## Supplementary Materials

The supporting information can be downloaded at: https://www.mdpi.com/article/10.3390/ijms242216395/s1.
