# Supplementary material for: Can Activation of Acetylcholinesterase by β-Amyloid Peptide Decrease the Effectiveness of Cholinesterase Inhibitors?
Source: Int J Mol Sci. 2023 Nov 16;24(22):16395. doi: 10.3390/ijms242216395 (PMC10671303; doi:10.3390/ijms242216395)
Supplement: Supplementary file 1 [file ijms-24-16395-s001.zip › ijms-2707148-supplementary.pdf]

Supplementary Materials

Can Activation of Acetylcholinesterase by β-Amyloid Peptide Decrease the Effectiveness of Cholinesterase Inhibitors?

Irina V. Zueva [1], Elmira A. Vasilieva [1], Gulnara A. Gaynanova [1], Andrey V. Moiseenko [2], Anna D. Burtseva [3,4], Konstantin M. Boyko [3], Lucia Ya. Zakharova [1] and Konstantin A. Petrov [1,5,*]

[1] Arbuzov Institute of Organic and Physical Chemistry, Federal Research Center "Kazan Scientific Center of the Russian Academy of Sciences", Arbuzov Str., 8, 420088 Kazan, Russia
[2] Faculty of Biology, Lomonosov Moscow State University, Leninskie Gory, 1–12, 119991 Moscow, Russia
[3] Bach Institute of Biochemistry, Research Center of Biotechnology of the Russian Academy of Sciences, Leninsky Prospect, 33/2, 119071 Moscow, Russia
[4] Landau Phystech School of Physics and Research, Moscow Institute of Physics and Technology, Institutsky Lane, 9, Dolgoprudny, 141700 Moscow, Russia
[5] Institute of Fundamental Medicine and Biology, Kazan Federal University, 18 Kremlyovskaya Str., 420008 Kazan, Russia
* Correspondence: kpetrov2005@mail.ru

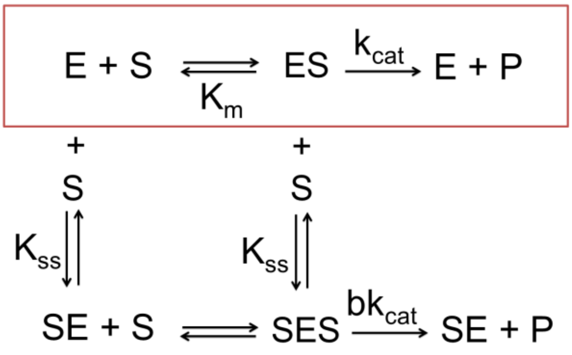

Scheme S1. Radic model. E – ChE; ES – complex of ChE with substrate; k_{cat} – first–order rate constant. Boxed part of the scheme is for hydrolysis at low substrate concentration. It corresponds to the Michaelis-Menten behavior with characteristic parameters: K_m and $V_{max}/(E) = k_{cat}$. Under conditions of excess substrate, a second molecule of substrate binds to the peripheral anionic site and affects the kinetics (bottom part of the scheme). A second molecule of substrate binds to the peripheral anionic site, giving a ternary complex, SES, characterized by a dissociation constant K_{ss}. This process leads to a change causing an alteration in the catalytic constant kcat by a b factor.
